# Effects of a health information system data quality intervention on concordance in Mozambique: time-series analyses from 2009–2012

**DOI:** 10.1186/s12963-015-0043-3

**Published:** 2015-03-26

**Authors:** Bradley H Wagenaar, Sarah Gimbel, Roxanne Hoek, James Pfeiffer, Cathy Michel, João Luis Manuel, Fatima Cuembelo, Titos Quembo, Pires Afonso, Victoria Porthé, Stephen Gloyd, Kenneth Sherr

**Affiliations:** Department of Epidemiology, University of Washington School of Public Health, 1959 NE Pacific Street, Seattle, WA 98195 USA; Health Alliance International, Seattle, WA USA; Department of Family Child Nursing, University of Washington, Seattle, WA USA; Health Alliance International, Beira, Mozambique; Ministry of Health, Beira Operations Research Center, Beira, Mozambique; Department of Global Health, University of Washington, Seattle, WA USA; Community Health Department, School of Medicine, Eduardo Mondlane University, Maputo, Mozambique

## Abstract

**Background:**

We assessed the effects of a three-year national-level, ministry-led health information system (HIS) data quality intervention and identified associated health facility factors.

**Methods:**

Monthly summary HIS data concordance between a gold standard data quality audit and routine HIS data was assessed in 26 health facilities in Sofala Province, Mozambique across four indicators (outpatient consults, institutional births, first antenatal care visits, and third dose of diphtheria, pertussis, and tetanus vaccination) and five levels of health system data aggregation (daily facility paper registers, monthly paper facility reports, monthly paper district reports, monthly electronic district reports, and monthly electronic provincial reports) through retrospective yearly audits conducted July-August 2010–2013. We used mixed-effects linear models to quantify changes in data quality over time and associated health system determinants.

**Results:**

Median concordance increased from 56.3% during the baseline period (2009–2010) to 87.5% during 2012–2013. Concordance improved by 1.0% (confidence interval [CI]: 0.60, 1.5) per month during the intervention period of 2010–2011 and 1.6% (CI: 0.89, 2.2) per month from 2011–2012. No significant improvements were observed from 2009–2010 (during baseline period) or 2012–2013. Facilities with more technical staff (aβ: 0.71; CI: 0.14, 1.3), more first antenatal care visits (aβ: 3.3; CI: 0.43, 6.2), and fewer clinic beds (aβ: -0.94; CI: −1.7, −0.20) showed more improvements. Compared to facilities with no stock-outs, facilities with five essential drugs stocked out had 51.7% (CI: −64.8 -38.6) lower data concordance.

**Conclusions:**

A data quality intervention was associated with significant improvements in health information system data concordance across public-sector health facilities in rural and urban Mozambique. Concordance was higher at those facilities with more human resources for health and was associated with fewer clinic-level stock-outs of essential medicines. Increased investments should be made in data audit and feedback activities alongside targeted efforts to improve HIS data in low- and middle-income countries.

## Background

National-level, ministry-led health information systems (HIS) are widely touted as a “foundation of public health,” [1] with available, reliable, timely, and valid data accepted as a prerequisite for decision-making and the provision of high-quality health services at all levels of the health care system. Published literature, however, is replete with studies detailing low quality of routine HIS data among many low-and middle-income countries (LMICs) [2-6]. In addition, failed attempts to use HIS data to monitor or evaluate the effects of health interventions or to conduct operational research are common [7-10].

Groups working in multiple LMICs have recently shown that rapid and effective methods for improving HIS data exist and have been tested [11]. In KwaZulu-Natal, South Africa, a seven-month data quality intervention consisting of three-day trainings, monthly data meetings, and data quality audits (DQAs) at health facilities increased data completeness from 26% to 64% and data accuracy from a correlation of 0.54 to 0.92 [12]. Interventions as simple as implementing quarterly data review workshops and fostering the use of HIS data for decision-making have resulted in improved data quality and coverage in diverse LMIC settings [13,14].

While case studies of short-term data quality interventions have been previously illustrated, no studies have quantitatively evaluated the relationship between health system factors and facility-level intervention effect heterogeneity over longer time periods. The objective of the present study is to measure the impact of a data quality intervention over three years and to identify factors associated with changes in HIS data concordance over time in Mozambique. Identifying these factors could improve the development and targeting of future interventions to improve HIS data in LMICs.

## Methods

### Study setting and data quality intervention

Funded through the Doris Duke Charitable Foundation’s African Health Initiative, the Mozambique Population Health Implementation and Training Partnership (PHIT) is a comprehensive public health system intervention focused in Sofala Province [15]. One key element of this intervention is to improve routine HIS data through continual assessment of the availability, consistency, and accuracy of HIS data. Beginning in 2010, annual DQAs have been conducted from a sample of 26 health facilities from all districts in Sofala Province. The study setting and profile of the 26 health facilities have been previously described [16]. In terms of the intervention, health facilities are publicly ranked by summary data concordance measures, and facilities with poor data quality receive additional supportive supervision and data training. Additional intervention components include: (1) district-level meetings bringing together front-line health workers and district/provincial managers for data feedback, performance gap identification, solution planning, and action plan monitoring; (2) the development and use of simple data dashboards for easy visualization of secular trends in key health indicators; (3) the development of simple human resource allocation optimization models; and (4) equipment purchase and maintenance. A full description of intervention components and an introduction to the Mozambican HIS have been previously published [15,17].

### Variable definitions and statistical analyses

#### Outcome of interest

Our outcome summarizes the availability and reliability (concordance) between a gold standard data quality audit and routine HIS data across four key indicators (outpatient consults, institutional births, first antenatal care visits [ANC1], and third dose of diphtheria, pertussis, and tetanus vaccination [DPT3]) and five levels of health system data aggregation (daily facility paper registers, monthly paper facility reports, monthly paper district reports, monthly electronic district reports, and monthly electronic provincial reports). As has been used in similar studies [12,18], data were deemed concordant if they had less than a 10% error margin comparing the gold standard DQA and routine HIS numbers. Each month’s value was compared for all five levels of data aggregation and across the four key indicators listed above and then averaged. That is, perfect facility concordance would be 16/16, representing four indicators multiplied by four comparisons across the five levels all achieving <10% error. If data were unavailable, concordance was zero for that indicator/level combination. DQA data teams consisted of trained data collectors external to the Ministry health system supervised by a data expert. Data were double-entered and managed in an Excel database. If there were discrepancies in abstracted DQA data, data collectors would validate their measurements by re-counting registry entries with the help of the expert supervisor.

#### Predictors of interest

Predictors were selected based on previous research regarding facility-level predictors of stock-outs of essential health products [16] and the realities of data availability. These included: type of health facility; health facility burden measured in number of outpatient consults or ANC1 visits; number of inpatient beds; number of technical staff (doctors, nurses, assistants); number administrative staff; distance from central drug and equipment distribution center; rural/urban location; and number of health facility drug stock-outs where the drug was available at the district-level drug depository. The relationship between stock-outs and data quality was evaluated for 2011 and 2012 only due to limited stock-out data availability. Detailed methods regarding data collection for drug stock-outs and other key predictors have been previously published [16].

### Analysis methods

Mixed-effects linear models were built in Stata 13 with 0-100% data concordance as our outcome of interest and α = 0.05 representing statistical significance using two-tailed tests. Our analysis plan included: (1) local regression across time and clinics to determine functional forms for variable parameterization; (2) crude analyses of data trends; and (3) analyses of each explanatory variable and its effect on data quality after accounting for the confounding effect of time using linear splines with yearly knots and random intercepts and slopes for clinics; and (4) fully-adjusted analyses controlling for time and simultaneous adjustment for all predictors. For all models, significance of group variables (health facility type, number of drug stock-outs) was determined by a chunk test prior to interpreting within-group associations. Analyses of residual plots indicated no significant lack of model fit at all steps.

### Ethics statement

This study was approved by the Mozambican National Institutional Review Board. The University of Washington deemed this study exempt as it focused on program evaluation purposes and was not considered human subjects research under United States federal regulations.

## Results

Descriptive statistics, the basic profile of health facilities surveyed, and information about the study setting have been previously published [14,15]. The intraclass correlation coefficient was 0.26 (confidence interval [CI]: 0.14, 0.37). Baseline median concordance in 2009 was 56.3% and concordance increased to 87.5% by 2012 (Table 1). There was no significant change in concordance prior to the data quality intervention (2009–2010). Concordance improved significantly by an average of 1.04% per month (95% CI: 0.60, 1.49) from 2010–2011 and 1.56% (CI: 0.89, 2.22) from 2011–2012 while the DQA intervention was implemented across health facilities. Intervention activities continued in 2012–2013, but no significant increase in data quality was observed.

**Table 1:** **Crude time trends in data concordance across 26 public-sector health clinics undergoing data quality intervention, 2009–2012, Sofala Province, Mozambique**

**Characteristic**	β* (95% CI)	**Model-fitted concordance (%)**	**Raw median concordance (%)**	**P-value**
*Monthly change in concordance*				
2009-2010	−0.237 (−1.00, 0.567)	53.5	56.3	0.54
2010-2011 (intervention began)	1.04 (0.596, 1.49)	59.0	62.5	<0.0001
2011-2012	1.56 (0.887, 2.22)	74.8	81.3	<0.0001
2012-2013	0.091 (−0.394, 0.577)	84.0	87.5	0.71
**Overall average monthly change**	0.877 (0.676, 1.08)	67.8	75.0	<0.0001

*β represents unadjusted monthly change in percent data concordance through use of a linear mixed model with random intercepts for clinics and random slopes over time. A linear spline was used to model time with knots at 12 (January 2010), 24 (January 2011), and 36 (January 2012) months of data collection.

Each 100-unit increase in first antenatal visits was associated with 3.3% higher (CI: 0.43, 6.2) data concordance, while each additional inpatient bed was associated with 0.94% (CI: −1.7, −0.20) lower data concordance (Table 2). Further, each additional technical staff at the health facility was associated with 0.71% higher (CI: 0.14, 1.3) data concordance.

**Table 2:** **Health facility factors associated with data concordance across 26 public-sector health clinics undergoing data quality intervention 2009–2012, Sofala Province, Mozambique**

**Characteristic**	aβ* adjusting for time only* (95% CI)	**P-value**	**aβ** ^**†**^ **fully-adjusted model (95% CI)**	**P-value**
Monthly number of 1st antenatal care visits (100 unit change)	2.8 (0.08, 5.6)	0.04	3.3 (0.43, 6.2)	0.02
Monthly number of outpatient visits (1000 unit change)	0.23 (−0.75, 1.2)	0.65	−0.12 (−1.2, 0.91)	0.82
Rural clinic location	13.7 (−12.5, 39.9)	0.31	40.1 (−9.7, 91.7)	0.11
Number of clinic beds	−0.10 (−0.39, 0.20)	0.52	−0.94 (−1.7, −0.20)	0.01
Distance from drug distribution point in kilometers	0.11 (−0.27, 0.49)	0.57	0.10 (−0.48, 0.68)	0.75
*Type of health facility* ^§^				
Rural health center – Type 1	1 (reference)		1 (reference)	
Rural health center – Type 2	12.2 (−8.0, 32.4)	0.24	−8.0 (−39.6, 23.6)	0.62
Urban health center – Type A	−0.56 (−34.3, 33.2)	0.97	30.8 (−27.1, 88.8)	0.30
Rural hospital	18.7 (−7.9, 45.4)	0.17	44.2 (0.91, 87.5)	0.05
*Clinic human resources*				
Number of technical staff	0.28 (−0.12, 0.67)	0.17	0.71 (0.14, 1.3)	0.02
Number of administrative staff	0.43 (−1.6, 2.4)	0.68	−0.86 (−3.3, 1.6)	0.50
*Number of clinic drug stock-outs with availability at district* ^‡^				
Zero drugs stocked out	1 (reference)		1 (reference)	
One drug stocked out	0.12 (−4.0, 4.2)	0.95	0.10 (−4.2, 4.4)	0.97
Two drugs stocked out	0.43 (−4.1, 5.0)	0.85	−0.63 (−5.4, 4.1)	0.80
Three drugs stocked out	3.9 (−8.5, 16.3)	0.54	2.3 (−9.6, 14.6)	0.70
Four drugs stocked out	−11.9 (−22.9, −0.99)	0.03	−10.0 (−20.7, 0.78)	0.07
Five drugs stocked out	−50.7 (−63.9, −37.4)	<0.0001	−51.7 (−64.8, −38.6)	<0.0001

*aβ represents the relationship between data concordance and key factors adjusting for the confounding effect of time through use of a linear spline in a linear mixed model with random slopes and intercepts across health facilities.

^†^aβ represents relationship between data concordance adjusting for time as above along with multivariate adjustment for all key predictors.

^‡^Association evaluated for years 2011 and 2012 only due to limited availability of stock-out data. Group variable significant at p < 0.001 for both models.

^§^Group variable not significant for model adjusting for time only (p = 0.45), or fully-adjusted model (p = 0.22).

The factor most strongly associated with concordance was the number of essential drugs stocked out at health facilities while the drug was available at the district headquarters. Compared to those clinics with no drug stock-outs, those with five drugs stocked out had 51.7% (CI: −64.8, −38.6) lower data concordance.

## Discussion

Similar to previous studies in sub-Saharan Africa [11-14], the present study found that an intervention consisting of data audits, equipment/supply purchase and maintenance, supportive supervision to low-performing clinics and feedback from district/provincial levels, data trainings, and district performance enhancement meetings focused on improving data use for decision-making can result in rapid improvements in data concordance in public-sector health facilities. Novel findings from our study in Mozambique are that: (1) improvements in data quality occur most significantly during the first two years and may hit a plateau of approximately 85-90% mean concordance; (2) improvements in data reliability can be sustained over multiple years given continued intervention activities; (3) higher numbers of human resources for health are associated with larger gains in data concordance; (4) facilities attending more antenatal care visits and those with fewer inpatient beds also show greater increases in concordance; and (5) stock-outs of essential medicines for primary health care provision are strongly associated with poor HIS data quality.

Our findings that data improvements were not related to determinants such as facility location (rural/urban, distance from district headquarters) and facility type are promising given that these more “static” infrastructure-related factors are difficult to modify in the short term. Given this, rapid and equitable data improvements appear possible even at rural peripheral health facilities that traditionally have the fewest health resources. These results support past evidence suggesting that management issues centered around motivation and value placed on the quality of routine data collection [14,19], as well as health worker numeracy and training [20], may be significant determinants of poor HIS data quality in LMICs. Our study builds on these previous findings by showing that, controlling for health facility location and type, interventions to improve data quality may be less effective at facilities with few human resources for health or large amounts of high-burden inpatient services. Further research should clarify how facility burden characteristics (number of ANC1 visits, outpatient visits) are related to data improvements because of our counterintuitive findings of a positive relationship between ANC1 visits and data concordance, but no corresponding association with outpatient visits.

Given that HIS data quality gains can be sustained over multiple years (allowing reliable data-driven decision-making), and that relatively simple data improvement interventions have been tested and shown effective in multiple LMIC settings, donors and governments should consider investments in DQAs and other interventions to improve routine data systems. These investments are especially important given recent analyses indicating potentially increasing subnational disparities in health statistics in LMICs [21] and the difficulty of traditional survey designs (Demographic and Health Surveys/Multiple Indicator Cluster Surveys) to provide health statistics below the provincial level [10,22]. Moreover, our findings further support the idea that quality HIS data are necessary for high-quality service provision, such as supply management of essential medicines and the forecasting of future supply needs to guard against stock-outs.

Our study has a number of limitations. First, without an adequate control group we cannot eliminate the possibility that all clinics in Mozambique are experiencing similar data improvements. Second, significant increases in data concordance do not necessarily mean that data validity has improved – a more difficult metric to evaluate. Third, the key indicators evaluated may not be representative of all HIS indicators essential for program planning and service provision. Last, the present study was conducted in one province of Mozambique and in a subset of clinics and therefore may not be representative of all public health clinics nationally.

## Conclusion

We found that an intervention consisting of facility-based data audits, targeted training and supervision, equipment purchase/maintenance, and data audit and feedback meetings was associated with significant increases in public-sector HIS data concordance. Improvements were greater at health facilities with more human resources for health, more antenatal care visits, and fewer inpatient beds. Given the importance of available, reliable, timely, and valid data for decision-making and health care provision – such as effective management of essential medicines – donors and Ministries of Health should consider increased investments in improving HIS data quality. Future studies should aim to identify which data quality intervention components are most effective and to determine the sustainability of data quality interventions over the longer term.
